# Experience of using telecare in carrying out a program of psychosocial rehabilitation of patients with schizophrenia and their relatives during the Covid-19 pandemic

**DOI:** 10.1192/j.eurpsy.2022.1935

**Published:** 2022-09-01

**Authors:** V. Mitikhin, T. Solokhina, M. Kuzminova

**Affiliations:** FSBSI “Mental Health Research Centre”, Department Of Mental Health Services, Moscow, Russian Federation

**Keywords:** rehabilitation, psychosocial, telecare, schizophrénia

## Abstract

**Introduction:**

During the Covid-19 pandemic, patients with mental illness turned out to be one of the most vulnerable groups of the population, since the forced self-isolation regime was a decrease in the availability of psychiatric care. During this period, the use of telemedicine increased to provide timely assistance.

**Objectives:**

To analyze the experience of telecare in program of psychosocial rehabilitation of patients with schizophrenia and their relatives and to evaluate its effectiveness.

**Methods:**

80 schizophrenia patients in remission of varying quality and 41 relatives participated in rehabilitation program. To assess the effectiveness of telecare, PANSS, SF-36, URICA, PHQ-9, ISI, PSS-10, GAD-7 scales were used.

**Results:**

Psychosocial interventions through telecare were carried out for 12 months. Patients and relatives participated in video sessions on Zoom and Skype Internet platforms, as well as in instant messengers. Rehabilitation program for patients included psychoeducation, skills training, art-therapy, music therapy, bibliotherapy, psychological counseling. Relatives were provided with psychoeducation and psychological counseling. The analysis showed that the use of telecare contributed to increase in the availability of psychotherapeutic assistance, the participation of patients with low motivation and prompt problem solving. Within the studied period, only 5% of patients (4 persons) developed relapses, two patients (2.5%) were hospitalized. Patients and relatives showed a high level of satisfaction with the care provided, positive dynamics of psychological indicators.

**Conclusions:**

The effectiveness of psychosocial rehabilitation program through telecare has been proven. The possibility of carrying out various psychosocial interventions in online format has been shown.

**Disclosure:**

No significant relationships.

